# The ANIBES Study on Energy Balance in Spain: Design, Protocol and Methodology

**DOI:** 10.3390/nu7020970

**Published:** 2015-02-04

**Authors:** Emma Ruiz, José Manuel Ávila, Adrián Castillo, Teresa Valero, Susana del Pozo, Paula Rodriguez, Javier Aranceta Bartrina, Ángel Gil, Marcela González-Gross, Rosa M. Ortega, Lluis Serra-Majem, Gregorio Varela-Moreiras

**Affiliations:** 1 Spanish Nutrition Foundation (FEN), c/General Álvarez de Castro 20, 1ªpta, 28010 Madrid, Spain; E-Mails: eruiz@fen.org.es (E.R.); jmavila@fen.org.es (J.M.A.); adrian26580@gmail.com (A.C.); tvalero@fen.org.es (T.V.); susanadelpozo@fen.org.es (S.P.); prodriguez@fen.org.es (P.R.); 2 Department of Preventive Medicine and Public Health, University of Navarra, C/Irunlarrea 1, 31008 Pamplona, Spain; E-Mail: jaranceta@unav.es or javieraranceta@hotmail.com; 3 Department of Biochemistry and Molecular Biology II, and Institute of Nutrition and Food Sciences, University of Granada (SPAIN), Campus de la Salud, Avda. del Conocimiento, Armilla, 18100 Granada, Spain; E-Mail: agil@ugr.es; 4 ImFINE Research Group, Department of Health and Human Performance, Technical University of Madrid, c/Martín Fierro7, 28040 Madrid, Spain; E-Mail: marcela.gonzalez.gross@upm.es; 5 Department of Nutrition, Faculty of Pharmacy, Complutense University, Plaza Ramón y Cajal s/n, 28040 Madrid, Spain; E-Mail: rortega@ucm.es; 6 Research Institute of Biomedical and Health Sciences, Universidad de Las Palmas de Gran Canaria, Facultad de Ciencias de la Salud, c/Doctor Pasteur s/n Trasera del Hospital, 35016 Las Palmas de Gran Canaria Las Palmas, Spain; E-Mail: lluis.serra@ulpgc.es; 7 Department of Pharmaceutical and Health Sciences, Faculty of Pharmacy, CEU San Pablo University, Urb. Montepríncipe, crta. Boadilla km. 5.3, Boadilla del Monte, 28668 Madrid, Spain

**Keywords:** energy balance, dietary intake, physical activity quantification, new technologies, Spanish food and dietary patterns, nutrition surveys

## Abstract

Energy Balance (EB) is an important topic to understand how an imbalance in its main determinants (energy intake and consumption) may lead to inappropriate weight gain, considered to be “dynamic” and not “static”. There are no studies to evaluate EB in Spain, and new technologies reveal themselves as key tools to solve common problems to precisely quantify energy consumption and expenditure at population level. The overall purpose of the ANIBES (“Anthropometry, Intake and Energy Balance”) Study was to carry out an accurate updating of food and beverage intake, dietary habits/behaviour and anthropometric data of the Spanish population (9–75 years, *n* = 2009), as well as the energy expenditure and physical activity patterns. Anthropometry measurements (weight, height, body mass index, waist circumference, % body fat, % body water) were obtained; diet was evaluated throughout a three-day dietary record (*tablet* device) accompanied by a 24 h-dietary recall; physical activity was quantified by questionnaire and accelerometers were also employed. Finally, information about perception and understanding of several issues related to EB was also obtained. The ANIBES study will contribute to provide valuable useful data to inform food policy planning, food based dietary guidelines development and other health oriented actions in Spain.

## 1. Introduction

At the present time, six out of the seven main risk factors for premature death in Europe (high blood pressure, inadequate blood cholesterol concentrations, increased Body Mass Index (BMI), insufficient intake of fruits and vegetables, physical inactivity, and alcohol abuse) are related to lifestyle, and more specifically, to poor dietary habits, and physical inactivity [1,2].

From a public health perspective, the increase in overweight and obesity is of particular concern in Europe [3] and more specifically in Spain [4]. Both, the World Health Organization (WHO) and the European Commission advocate for an integral approach. A good example of this is the recent *Vienna Declaration on Nutrition and Non Transmittable Diseases (2013)*, in the setting of the “Health 2020” Program [5].

The rapid social and lifestyle changes that have occurred in the last decades have led to a progressive abandoning of the traditional profile characteristic of the ‘Mediterranean lifestyle’ in Spain [6,7,8,9,10,11,12,13]. Technological advances and improvement of the socioeconomic conditions are closely linked to this transformation, namely better acclimatization conditions in the houses and workplaces, the mechanization of labor tasks, the improvement in public transportation, a great increase in the use of private motorized transportation, *etc.* Important changes in leisure time activities have notably contributed to increased sedentary time and reduction in the amount of physical activity [4]. Energy consumed during physical activity is the component that varies the most in the total energy expenditure, which is divided into exercise (planned activity), and non-exercise thermogenesis (NEAT; this would include daily living activity) [14,15].

The availability of detailed and high quality food consumption and physical activity data is essential to carry out public health nutrition initiatives in Europe [16,17,18,19,20]. Methodologies and procedures used in dietary surveys have mainly been developed with the aim of evaluating the nutritional status of a population, *i.e.*, the intake of energy, macronutrients and/or micronutrients. However, the problem of underreporting is consistent in different surveys and therefore the use of new methodologies to avoid usual bias is challenging [21,22,23,24,25,26,27]. The possibility for “real-time” recording at eating events is not based on manual selection from pre-defined food items, but rather on digital photography or voice recording [28,29,30,31,32]. Moreover, there is a consensus that diet and food composition and consumption still is a large unknown in many of its aspects, and even more as it has currently become more complex [16,17,33].

*Energy Balance* (EB) is defined as the state achieved when energy intake equals energy expenditure and is considered to be “dynamic” and not “static” [34]. Although an imbalance in energy consumption and expenditure is required to promote inappropriate weight gain, the relative contributions of each to obesity remains under debate [35]. Integral studies of all the elements comprised in the EB equation should be made given their interrelationship [36].

It seems essential to improve the tools for studying the energy intakes and losses of “free living” independent subjects. In this regard, tools such as databases of the composition of quality foods, especially regarding energy and serving sizes, should be improved, as clearly stated at the recent (2013) Consensus Document and Conclusions on “Obesity and Sedentarism in the 21st Century: What can be done and what must be done?” [4].

Different valuable dietary surveys have been conducted in Spain, although to the best of our knowledge, no one has attempted to specifically approach EB. Briefly, the first Food Consumption Survey was performed in 1956 under the National Health Survey. Further, several Spanish Food Consumption and Nutrition Surveys have been carried out (ENNAs; 1964–1965, 1980–1981 y 1990–1991) mainly in collaboration with the National Statistics Office (INE, Spain) [37,38,39,40]. From 1987 onwards, the current Ministry of Agriculture, Food and Environment (MAGRAMA) in Spain launched the National Food Consumption Survey (Panel), for which the Spanish Nutrition Foundation (FEN) is responsible for analyzing the dietary patterns and energy/nutrient intake of the Spanish population from the year 2000 onwards [6,41,42]. AECOSAN (Spanish Agency for Consumer Affairs, Food Safety and Nutrition) recently carried out the ENIDE Survey (Encuesta Nacional de Ingesta Dietética) (AECOSAN, 2012) [43]. At present, the so-called ENALIA (Encuesta Nacional de Alimentación en la población Infantil y Adolescente) Survey in children and adolescents from Spain is being carried out also under the auspices of AECOSAN. The latter updates the reference survey in Spain in children and young people (2–24 years old) called EnKid [44], and the AVENA study, a multicenter nutrition survey in Spanish adolescents [45]. At the regional level, other valuable and representative surveys have been conducted: The Region of Madrid [46] which has been recently updated by FEN [47], Catalonia [48], the Region of Valencia [49], Galicia [50], and Basque Country [51], among others. However, when approaching the other main EB determinant (“energy expenditure”) studies are much less frequent. The National Health Survey in Spain (2013) [52] revealed that 41.3% of the adult Spanish population is considered as sedentary, higher for women (46.6%) than for men (35.9%). Considering both their main and their leisure time activity, 40.9% of the adults (49.4% males, 32.4% females, aged 15–69 years) perform strenuous to moderate weekly physical activity. There is consensus at present that not only physical activity level but also inactivity and/or sedentary behavior should be taken into account and quantified [15,53,54].

The present ANIBES (“Anthropometry, Intake, and Energy Balance in Spain”) study, for which the design, protocol, and methodology are fully described in the present article, aims at adding new scientific-based evidence to describe the interplay among energy intake, energy expenditure, and body energy stores and how an understanding of EB must be considered as a useful tool either at the individual or population level.

### 1.1. Goals

The main goal of the ANIBES Study was to evaluate energy intake and energy expenditure in a national representative sample of the Spanish population by using innovative methodological tools in order to approach the EB concept. In addition, body composition and different dietary patterns and dietary quality indexes were also evaluated.

### 1.2. Specific Goals

- To determine total energy intake in the Spanish population aged 9–75 years, and its distribution by age group and sex.
- To determine total energy expenditure in the Spanish population aged 9–75 years, stratified by age group and sex.
- To analyze the main food groups and subgroups contributing to energy intake and differences by age group and sex.
- To evaluate different anthropometric measurements of special interest for the *energy balance* hypothesis.
- To describe the perception and understanding of different items in relation to EB for the Spanish population.

## 2. Experimental Section

### 2.1. Pilot Studies

The final fieldwork was carried out from mid-September to November (three months) 2013, but two pilot studies were previously carried out, as follows:

Once the methodology was developed, a first pilot study was carried out in June 2013. For this purpose, 2060 individuals were contacted: 162 (7.8%) agreed to have the first visit/interview, 142 participated at the second visit/interview, but only 97 were able to make the three-day dietary record by using the tablet. Finally, only 57 participants were considered as fully eligible. Therefore, a high rate of non-responders was observed mainly in the older age groups and parents of children and adolescents. The first pilot study allowed reviewing several issues, both software and questionnaires.

Once the results from the pilot study were completed, several *working/discussion groups* were created in order to improve the study design, protocols, software and manuals. Therefore, four groups (one of interviewers; two mixed groups of young adult people from 25–35 years old; one group of parents with children aged 9–17 years) worked in order to improve the deficiencies observed during the fieldwork.

A second pilot study was carried out in order to evaluate the improvements after the first pilot study and comments and recommendations from the working groups. A total of 60 subjects (52 used tablet device; 5 photo camera; 3 by phone interview) participated. The second pilot study demonstrated the efficacy of the amendments made and validated the tools and questionnaires to be used later during the main fieldwork of the ANIBES Study.

### 2.2. Study Design and Sampling Procedure

The final protocol was approved by the Ethical Committee for Clinical Research of the Region of Madrid (Spain). The study was coded as “FEN 2013”, and approved on 31 May 2013.

The ANIBES Study aimed a sample size which should be representative of all individuals living in Spain (excluding the autonomous cities of Melilla and Ceuta) aged 9–75 years, living in municipalities >2000 inhabitants.

The sample for the ANIBES Study was designed based on 2012 census data published by the INE (Instituto Nacional de Estadística/Spanish Bureau of Statistics) for Gender, Age, Habitat Size and Region. Individual quotas were defined for each of these variables, which allow the identification of the total numbers of interviews required to properly represent the socio-demographic distribution under study. In addition, interlocking quotas were established for Age within Region and Habitat size within Region to ensure that the diversity of the population within each region was properly represented. The combination of individual and interlocking quotas provided the number of interviews to be achieved on each quota cell. The sample design and the high number of sampling points determined the number of required interviewers and their interviewing area. Each interviewer was given an exact number of interviews by age and gender within his or her sampling area to ensure the representation of the different demographics within the area. The sample selection procedure based on random routes eliminated any potential bias originated by proximity or familiarity of respondents. Table 1 shows the expected number of interviews based on the 2012 Census, as well as the final number of interviews achieved in ANIBES. The total sample size was calculated based on a 0.05 probability of Type I error (rejecting a null hypothesis when it is true) and 0.1 probability of Type II error (accepting a null hypothesis when it is wrong) in the main outcome of the study (energy intake).

The initial potential sample was 2634 individuals, and the final sample was 2009 individuals (2.23% error and 95.5% confidence interval). In addition, for the youngest groups (9–12, 13–17 and 18–24 years old), a boost was considered in order to have at least a *n* = 200 per age group and increase the statistical power of the study (error +/−6.9%). The booster interviews are only analysed in the context of the analysis of these specific subgroups and not in the context of the analysis of the main random sample. Therefore, the final random sample plus booster was 2285 participants.

The ANIBES sample was comprised of 50.4% of males and 49.6% females. The sample reflected the distribution in the population living in Spain. A more detailed description of the sample for the ANIBES study is shown in Table 1.

**Table 1:** Distribution of the sample for the ANIBES study.

	Base	Sample (*n*)		
		Initial targeted Sample	Final Sample	Final + Boost
		2634	2009	2285
sex	Men	1309	1013	1160
	Women	1325	996	1125
age (years)	Infants 9–12	240	100	213
	Adolescents 13–17	246	124	211
	Adults 18–64	1911	1588	1655
	Elderly 65–75	237	197	206

The sample quotas according to the following variables were:

- Age groups (in years): 9–12, 13–17, 18–64 and 65–75.
- Gender: men and women.
- Region: seven Nielsen areas (Northeast, Levant, South, West, North Central, Barcelona, Madrid) and Canary Islands.
- Habitat size: 2000 to 30,000 inhabitants (rural population); from 30,000 to 200,000 inhabitants (semi-urban population) and over 200,000 inhabitants (city/town population).
- Other factors that were considered: rate of unemployment; % of foreigners (immigrant population), level of physical activity, and education/economical level.

**Figure 1:**
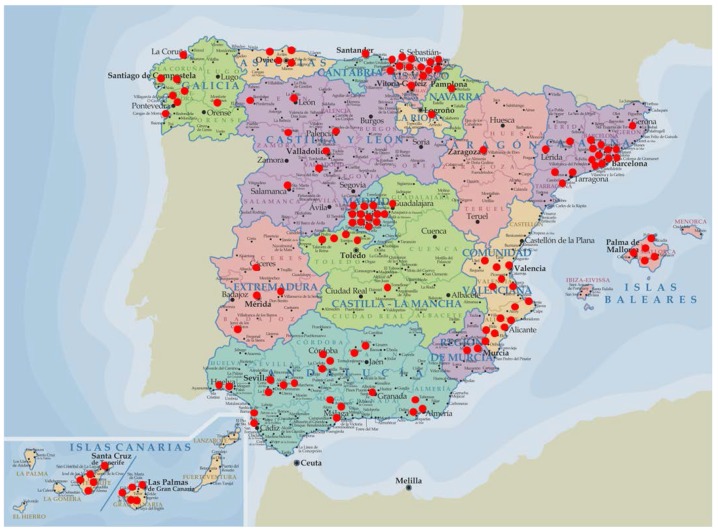
Geographical distribution of the sampling points for the ANIBES study.

The study was conducted through a stratified multistage sampling and for more coverage and representativeness, 128 sampling points were used, with 90 interviewers allocated in 11 areas and 12 coordinators, all previously trained by the *Spanish Nutrition Foundation* (FEN) (Figure 1). No previous pre-recruitment was considered, which minimized the risk of bias in responses [55].

### 2.3. Final Fieldwork

According to the number of interviews to be potentially targeted at the sampling point, one or more random initial routes for the sampling process were considered. The later criteria were not used for municipalities over 100,000 inhabitants where a postcode proportional criterion was considered. In the initial route, the apartment building or family housing was randomly selected, as well as the first household to be approached. Non-eligible addresses include vacant or derelict properties and institutions. If the *uptake* was positive, limits to be considered for a potential participant were:

Apartment building:

- 1–10 units, only one potential participant.
- 11–20 units, two potential participants as maximum.
- 21–50 units, three potential participants maximum.
- >50 units, four participants.

For family housing, one possible participant per 10 units was the rule used.

The survey was designed in order that no more than one adult and one child were selected from a household. This meant that adults living in households with one or more adults, and children in households with one or more children were less likely to be selected than were adults or children in single adult/child households.

Since one of the main drawbacks from the pilot studies was the initial rejection for “door-to-door” uptake, special efforts were made for better results at the main fieldwork: different informative posters about ANIBES goals were posted in the área/neighborhood, followed by letters that were sent to all the neighbors. In addition, during the first visit by the interviewer, an informative letter from the PI plus a leaflet and a set of infographics explaining the whole process were offered. Finally, the potential participant was informed about a small incentive (30 euros) for participation and a detailed final report including anthropometric data, physical activity level, and dietary/nutritional status, with an estimated value of 40–50 euros.

All interviewers, call center agents, and dieticians-nutritionists working on the ANIBES study were briefed and trained before undertaking an assignment and were monitored during their assignment. All interviewers attended a two-day training course designed by the FEN where they were fully briefed on the protocols and administration of the survey. Fieldworkers were also issued with comprehensive written instructions covering survey procedures and measurement protocols. The briefing sessions covered background and content, doorstep approach, questionnaire administration (including practice sessions), placement and collection of self-completions and ActiGraphs and the placement, checking and collection of the three-day food tablet diaries and 24 h-dietary recall and training in anthropometric data collection.

### 2.4. Stages

In order to cover a broad range of dates and to optimize the devices to be used during the study, several stages were designed, and comprised of:

#### 2.4.1. Stage 1: The Interviewer Visits

A letter and leaflet describing the purpose of the survey were previously posted in a potential targeted apartment building/family housing at the sampling points. A few days later, interviewers visited the addresses to determine whether the address was private, residential and occupied. They then carried out the selection process as previously explained.

The different days of the week would (as far as possible) be equally represented as each cycle always included two working days (Monday and Tuesday or Thursday and Friday), and one weekend day (Saturday for Thursday and Friday cycle or Sunday for Monday and Tuesday cycle).

**Figure 2:**
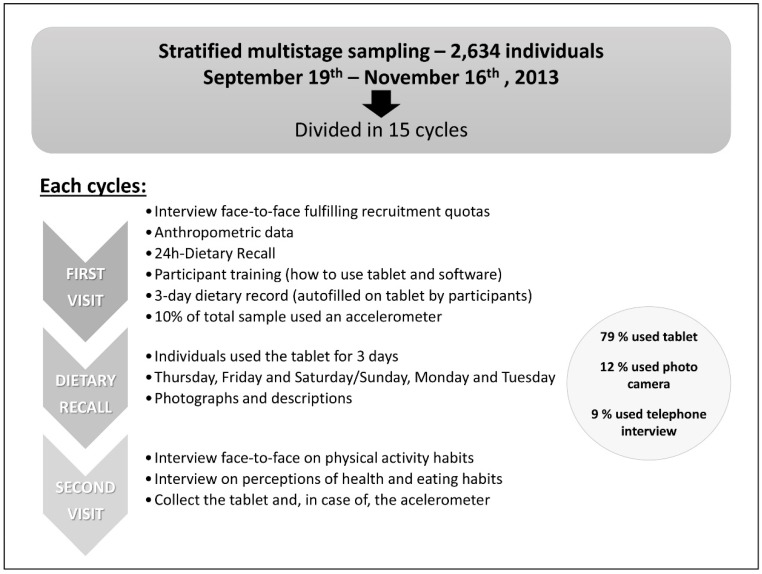
Scheme of main fieldwork of the ANIBES Study in Spain.

Interviewers carried out two main visits to households who agreed to participate (Figure 2):

The **first visit** (“face-to-face”) with an approximate duration of 60 min comprised the following items:

(a) Identification of the trained interviewer, as a collaborator of the FEN. The interviewer explained the main goals of the study, the design and stages, the novelty of the tools to be used for collecting food intake and recording physical activity, as well as offered to have a feedback report at the end of the study that included main results, dietary and physical activity advice, *etc.* The potential participant also received a letter from the principal investigator of the ANIBES Study, and was informed about the stipend for participation in the study. At this point, the potential participant was asked to sign the letter of consent for participation in the study.
(b) Inclusion/exclusion questionnaire: the interviewer verified through a filter questionnaire that the participant was eligible for the ANIBES Study. Several exclusion criteria were applied:
  - Those individuals living in an institution (e.g., colleges; nursing homes, hospitals, *etc.*)
  - Individuals following a therapeutic diet due to a recent surgery or any medical prescription.
  - If they were suffering a transitory pathology (*i.e.*, flu, gastroenteritis, chicken pox, *etc.*) at the time of the fieldwork.
  - Individuals employed in areas related to consumer science, marketing or the media.

However, individuals under the following conditions were considered eligible to be included:

- Those following dietary advice such as for prevention of hypertension, diabetes, hypercholesterolemia or hyperuricemia.
- Pregnant and lactating women.
- With diagnosed allergy and/or food intolerance.
- Suffering a metabolic disease such as hyperthyroidism or hypothyroidism.

(c) Anthropometric measurements: the trained interviewer collected the different measures following the procedures tested before at the two pilot studies:
  - Height: by triplicate using a Stadiometer model Seca 206 *(*Seca, Hamburg, Germany*).*
  - Weight: one determination in a weighing scale model Seca 804 (Seca, Hamburg, Germany*)*. This scale provide information about body mass index (BMI), percentage of body fat and percentage of body water.
  - Waist circumference: by triplicate using a tape measure model Seca 201 (Seca, Hamburg, Germany*)*. The volunteer was asked to stand and placed the tape measure around his/her middle, just above the hipbones. Measure was taken just after breathing out.
(d) 24-h Dietary Recall: no prior notification was given to the subjects about whether or when they would be interviewed about their food intake. An *ad hoc* questionnaire was designed and previously checked and modified at the already explained pilot studies. The participant recalled the food intake for the past 24 h. Food quantities were assessed by using of household measures, food models, pictures, or the brands. The 24-h dietary recall was designed for further verification of the information collected at the Tablet, but also to make the participant more familiar with the type of information to be recorded during the three-day period.
(e) Tablet device for collection of dietary data: the three-day food diary: All the participants were provided with a tablet device (*Samsung Galaxy Tab27.0*.) and instructed on how to record by taking pictures of all foods and beverages consumed, both at home and outside. Pictures had to be taken before and after finishing the meals. Additionally, a brief description of the meals, recipes, brands, *etc.* had to be also recorded with the device. The tablet was designed only to be used to collect information related to the ANIBES Study and no other uses were allowed.

A toll-free telephone number attended by call center-trained operators, was available for the participants in order to answer any questions about the software, use of the device, food and beverage record, *etc.* A manual of procedures to facilitate food collection was also given to the participants. Participants were also informed that insurance would cover any accident or incidence with the devices, although they were asked for correct and watchful use and maintenance.

(f) If the participant declared or demonstrated that he/she was unable to use the tablet device, other possibilities were offered: photo camera plus paper or telephone interview.
(g) At the end of the first visit, the date for the second interviewer visit was agreed, as well as the telephone calls to be made for check up at the end of the collection of the data.
(h) Accelerometer device to quantify physical activity level: The physical activity measurements was obtained with an accelerometer ActiGraph (model GT3x y GT3x+; *ActiGraph*, *Pensacola*, *FL*, *USA*). This provided a measure of the frequency, intensity, and duration of physical activity and allowed classification of activity levels as sedentary, light, moderate and vigorous. Individuals were asked to wear the ActiGraph on a belt above the right hip, during three consecutive full days including its cycle of the three days food and beverages diary record by the tablet.

Objective measurements of physical activity were taken using the ActiGraph, which recorded vertical movement, where the number of movement (“counts”) increased with the intensity of activity. For any individual, the accelerometer recorded different periods during the day spent at different levels of activity, *i.e.*, differing levels of “counts per minute” (cpm), while they were being sedentary or engaging in light, moderate, or vigorous activity. This provided a measure of the frequency, intensity, and duration of physical activity and allowed classification of activity levels as sedentary, light, moderate and vigorous. Individuals were asked to wear the accelerometer on a belt above the right hip, during waking hours for three consecutive full days in parallel with the three days of food and beverages diary record by the tablet. For the present study, the minimum wear time criterion for inclusion in analysis was set at three days. The average daily cpm for each participant was calculated as a weighted average based on the probability of wear/non-wear (for a minimum wearing time of at least eight hours per day). The participants were also provided with a sheet to be filled in with the periods (hours/minutes) of non-wear (shower, swimming, *etc.*).

For those participants that agreed to wear the accelerometer (*n* = 206) to quantify the physical activity, the device was activated coincident with the Tablet-based three-day food diary. The subsample was selected following the same criteria for representativeness as for the total sample included in the study.

After collecting the accelerometer devices at the second interviewer visit, they were sent to IPSOS in order to download the recorded information from the participant (physical activity, but also additional data such as sex, date of birth, height, and weight) and to recharge the battery for the next participant. The recorded information by the accelerometer in the subsample (167 adults and 39 children) was further used to validate the physical activity questionnaire administered to the whole sample, and to build a mathematical model to quantify energy expenditure in combination with different standard formulas.

The **second visit** (“face-to-face”) with also an approximate duration of 60 min comprised the following items: detailed interview about physical activity (International Physical Activity Questionnaire (IPAQ) for children and adolescents modified according to the HELENA study for children and adolescents [56]), and for adults [57]; detailed interview by using validated questionnaires previously tested at the pilot studies, designed to gain insights from the participants on important, nutrition and health-related topics mainly associated with EB, was also scheduled. Finally, the tablet device and the accelerometer were collected.

In this sense, energy expenditure (EE) in the ANIBES Study was collected using complementary measures by means of an objective (accelerometer) and a self-report (IPAQ) method. In the accelerometer subsample, EE was calculated as the sum of resting metabolic rate, RMR (Harris and Benedict formula) [58] and physical activity (Freedson formula for children [59], and for adults [60]). For estimating EE in the whole sample, accelerometer data, IPAQ, body composition and other related variables have been used to build up a statistical model with a set of significant and explanatory variables. By means of a STEPWISE process, those variables were chosen which best adjust to the dependent variable avoiding multicollinearity (strong dependencies between them). A statistical model was built in which the dependent variable is the real energy expenditure (provided by the accelerometer device), and the independent variables are other information coming from the physical activity questionnaire and anthropometric data. Model equation shows a good level of adjustment (*R*^2^ = 0.71) for the accelerometer subsample. Once this model has been built and validated at the accelerometer subsample, it extrapolated the energy to the rest of the whole sample based on the model equation as all the independent variables were available.

## 3. Results

A summary of the ANIBES fieldwork was as follows:

- Fieldwork dates: 19 September through 16 November 2013, structured in 15 different cycles/stages.
- 90 interviewers and 12 coordinators.
- Equipment:
  ○ 426 Tablet devices
  ○ 90 devices for anthropometric measurements (weighing scales, stadiometers, tape measures).
  ○ 87 accelerometers.
- Percentages of users for the different devices to collect food and beverages information was:
  ○ 79% of the sample used a Tablet
  ○ 12% used photo camera
  ○ 9% used telephone interview

A more detailed distribution of the devices used by sex and age group is shown in Table 2.

### 3.1. Data Processing

**Figure 3:**
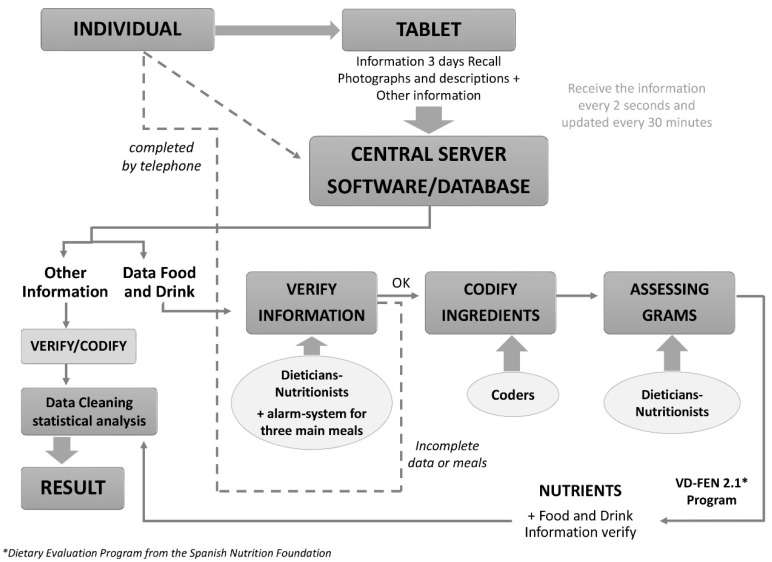
Main steps for codification and cleaning of data in the ANIBES Study. FEN= Spanish Nutrition Foundation

A detailed description of the whole process is shown in Figure 3.

The innovative technology used in this study allowed that the collected information could be verified and codified in nearly real time*.*

Participants also recorded for each eating occasion: where they were, who they were eating with and what they were doing at the time of eating/drinking. They also recorded if their intake was typical for that day (and if not, the reason why) and details of any dietary supplements taken. The software also contained a series of questions about usual eating habits (for example, type of milk or fat spread usually consumed) to facilitate coding.

**Table 2:** Devices used according to sex and age groups for the ANIBES study.

		SAMPLE											
		Initial Targeted Sample				Final Sample				Final Sample + Boost			
		Device				Device				Device			
		Base	Tablet	Photo Camera	Telephone	Base	Tablet	Camera	Telephone	Base	Tablet	Camera	Telephone
Sex	Base	2634	2077	320	237	2009	1568	253	188	2285	1804	279	202
		*100%*	*100%*	*100%*	*100%*	*100%*	*100%*	*100%*	*100%*	*100%*	*100%*	*100%*	*100%*
	Men	1309	1038	156	115	1013	800	124	89	1160	922	143	95
		*50%*	*50%*	*49%*	*49%*	*50%*	*51%*	*49%*	*47%*	*51%*	*51%*	*51%*	*47%*
	Women	1325	1039	164	122	996	768	129	99	1125	882	136	107
		*50%*	*50%*	*51%*	*51%*	*50%*	*49%*	*51%*	*53%*	*49%*	*49%*	*49%*	*53%*
Age (years)	Base	2634	2077	320	237	2009	1568	253	188	2285	1804	279	202
		*100%*	*100%*	*100%*	*100%*	*100%*	*100%*	*100%*	*100%*	*100%*	*100%*	*100%*	*100%*
	Children 9–12	240	201	29	10	100	82	15	3	213	178	27	8
		*9%*	*10%*	*9%*	*4%*	*5%*	*5%*	*6%*	*2%*	*9%*	*10%*	*10%*	*4%*
	Adolescents 13–17	246	221	21	4	124	113	8	3	211	190	18	3
		*9%*	*11%*	*7%*	*2%*	*6%*	*7%*	*3%*	*2%*	*9%*	*11%*	*6%*	*1%*
	Adults 18–64	1911	1571	207	133	1588	1300	176	112	1655	1361	180	114
		*73%*	*76%*	*65%*	*56%*	*79%*	*83%*	*70%*	*60%*	*72%*	*75%*	*65%*	*56%*
	Elderly 65–75	237	84	63	90	197	73	54	70	206	75	54	77
		*9%*	*4%*	*20%*	*38%*	*10%*	*5%*	*21%*	*37%*	*9%*	*4%*	*19%*	*38%*

Food and beverage records were returned from the field in real time to be coded by trained coders and editors. For this purpose, an *ad hoc* Central Server software/database was developed by IPSOS (Java/IE10 compatible) in order to be able to work in parallel with the verification process followed by the codification. The ANIBES software received the information from the field tablets every two seconds, and updated it every 30 min. The Central Server contained different modules to verify the information at the individual level but also according to the specific cycle; food weight and intakes; food codification and the assigning weight in grams. If for any reason, the terminal was unable to be connected to the network, the recorded information by the participant was saved, and resubmitted once the problem was solved. Finally, 189,600 inputs (ingredients) were managed from the 2009 participants, about 73 items per participant, and 24.3 food/beverages items per person/day as mean.

Coders attempted to match each food or drink item recorded in the tablet device with a food/portion code. For composite items which could be split into their component parts, each individual component was assigned. If an item had been recorded and there was no suitable code or there was insufficient detail to code the food, the entry was flagged as a query. Each food code is linked to appropriate portion size descriptors, such as a tablespoon for rice or pasta, which are then linked to the correct weight for that descriptor. So if a participant recorded/described their food using household measures, coders under dieticians-nutritionists checking would be able to select the appropriate portion size. If the portion size was described as a weight, the weight was entered directly into the system. Where the coder could not resolve the food or portion consumed, the entry was flagged as a query for action by a researcher who had greater nutrition knowledge and experience. The dieticians-nutritionists assigned appropriate codes for all flagged food and portion codes and checked any other queries raised by the coders. In general, where details for the coding of foods were missing, formally agreed default codes were used. Where portion sizes were missing, an estimate was made using the same weight if the food was consumed on another dietary day, or a portion size consistent with the participant’s usual consumption (e.g., small, medium or large), or an age-appropriate average portion.

For new products not included in the software, supermarkets or retail markets were visited or the manufacturer contacted to obtain information on nutrient content in order to decide whether a new food code was needed. This decision was based on nutritional composition compared to that of existing codes, as well as the frequency of consumption. If a new food code was required, the nutrient content was entered into the database. In the case of school meals, school caterers information about the nutrient content and portion size of dishes was considered.

### 3.2. Quality Control

The quality control of the collected information was supervised by trained dieticians-nutritionists, according to the following protocol:

(a) The same dietician-nutritionist was responsible for checking the food records included by the participant during the three-day dietary food record study.
(b) The initial quality control was based on the photographs and descriptions sent by the participants, but also the brief description that was asked before/after each meal and/or intake. Special care was given to validate some variables such as ingredients, brands of the processed and ready-to-eat foods, portion size or culinary technique in order to obtain accurate information for further codification.
(c) The final approval of the received information was given by a dietician-nutritionist and supervisor.

It is also of importance that the software used had an alarm-system when no records from the different three main meals were available.

At the start of the coding process, dieticians-nutritionists worked together with the coders checking the information and giving them individual feedback on their work (food and portion code entries). Portion code errors (selecting the wrong portion size descriptor or entering an incorrect weight) were more common than selecting the wrong food code. Where errors were found they were corrected. All of the entries flagged as a query by the coders were categorized into different query types, such as food code or portion code not available in the used software, recipes, missing or insufficient detail to code food or portion. Final quality checking was performed using each participant’s mean energy and nutrient intake (all reported nutrients) over the food and beverages diary record period (three days). Extreme intakes were considered from the mean and all entries in this region were checked against the diary.

Intakes of nutrients were calculated from the food consumption records using a special adapted VD-FEN 2.1(Dietary Evaluation Program from the Spanish Nutrition Foundation) software for the ANIBES Study. All data were carefully evaluated before being incorporated into the VD-FEN ANIBES Database, and briefly stratified as:

○ Level 1–16 food and beverage groups;
○ Level 2–29 food and beverage subgroups;
○ Level 3–761 food and beverages entries;
○ Company and brand;
○ Culinary treatment;
○ Household measure (tablespoons, glasses, cups, plates); typical/most used portion sizes and recipes from Spain; or conventional units/measures (e.g., 1 yogurt, 1 apple piece, half tomato, 1 slice of bread, 1 soda can, 1 biscuit, butter portion, *etc.*).

### 3.3. Data Cleaning

Once the data from the Tablet devices were coded and transferred into the ANIBES Database, a data cleaning process was performed:

First data cleaning stage: Participants were considered as fully eligible if after a cautious review of the information, it was verified that the three days were recorded using the tablet. Where registers were above or below the three-day established period, the following criteria was adopted:

- If a participant only had records from two or less days, he/she was considered as not valid and eliminated from the final sample.
- If a participant was registered four or more days, valid data were for those three collected days corresponding to the specific cycle of the participant, but always under the same scheme: 2 working days + 1 weekend day.

Second data cleaning stage: Participants were removed from the final sample if:

− Unexplained behavior in energy intake and large intra-individual variations between days were observed. When the known meal pattern of the participant was 3–5 intakes per day, but missing data was clearly observed in the register (*i.e.*, only breakfast and/or one meal per day), he/she was removed from the final sample.
− When extremely low energy intakes were recorded:
  ○ Less than 500 kcal/day in two or three days of the period.
  ○ Less than 500 kcal/day in one day, and <800 kcal/day in the remaining days.

Third data cleaning stage: Participants were considered valid if they fulfilled the following criteria:

- Having fulfilled previous data cleaning stages.
- Having completed successfully both visits during the fieldwork.
- If the participants had valid data on: weight, height, waist circumference.

Once all data had been verified, cleaned up, and approved, the ANIBES Database was developed. Calculation of energy and nutrient intakes was performed by the VD-FEN 2.1 Dietary Evaluation Programme from the Spanish Nutrition Foundation, mainly based on the Food Composition Tables (Moreiras *et al.*, 15th ed., 2011) [61], with several expansions and updates.

## 4. Discussion

The ANIBES Study (Antropometría, Ingesta, y Balance Energético en España; Anthropometry, Dietary Intake and Energy Balance in Spain) was designed to obtain accurate and updated information on the main determinants of the Energy Balance concept in Spain. For that purpose a country representative survey has been approached, and new technologies for dietary food record and physical activity level have been introduced for the first time in Spain. Anthropometry and information on dietary patterns and behavior as well as insights from the Spanish population on important, nutrition and health-related topics associated with EB were also obtained. Hereafter the design, protocol and methodology are discussed.

The sample size of a dietary survey per country is generally considered dependent on the variability of dietary consumption within each country. Information on this variability at a national/regional level is therefore needed in order to determine the number of subjects to include in a survey. We were able to collaborate with a renowned specialist such as IPSOS Spain for sample calculation, which guaranteed the procedure followed during the ANIBES Study. In addition, the accuracy of estimation of high consumption levels (percentiles 95th, 97.5th and beyond), which are key elements in dietary assessment, was also a priority in our survey. Percentiles calculated on a limited number of subjects bear large uncertainty, and are likely to provide biased estimations. According to Kroes *et al.* [62], high percentiles (*p*) can be assessed with sufficient accuracy if the sample size *n* satisfies the rule *n* (1 − *p*) ≥ 8, as scheduled during the ANIBES sampling design. In light of these considerations, the “Expert Group on Food Consumption Data” (EGFCD) in its recent document “*General principles for the collection of national food consumption data in the view of a pan-European dietary survey*” from EFSA [16], recommends for each country in Europe the inclusion of at least 260 subjects, 130 males and 130 females, in each of the six age classes identified (Infants, Toddlers, Other children, Adolescents, Adults and Elderly), which would lead to a minimum of 1690 participants per country according to the EGFCD. Since large countries are likely to show a variety of consumption patterns in the different geographical areas, they require the inclusion of additional subjects. The inclusion of further information on study participants (such as residence in regions, residence in urban or rural areas, residence in seaside or countryside, the type of habitat or the number of individuals belonging to the household) must also be considered during the sampling phase as already explained in the ANIBES design. However, a lower number of subjects could be acceptable for the infant group since their variability in consumption is potentially lower than for other population groups. Based on the above calculations and in line with estimates in the European Food Consumption Survey Method (EFCOSUM) project [63], the EGFCD [16] suggests that a minimum of 2000 subjects are sampled from each country to represent the total population at the national level. As for the ANIBES Study, the initial number of recruited participants (those who accepted to participate in the project, were administered the first visit interview and anthropometric measurements and were given the tablet to record intakes) was 2914. Out of these, 2634 (90.3%) recorded at least three days of intakes and responded to the second visit interview. Once all supervision and cleaning procedures were applied, the number of valid participants was 2285 (78.4% of the initial sample). The response rate in the national dietary surveys is generally low, ranging between 33% in Italy [64] and 70% in The Netherlands [65], which partly reflects a general trend towards lower response rates over time. In addition to choosing data collection methods with a lower burden for the respondents (the Tablet device was potentially considered to be attractive), also other kind of measures to keep the participation rate as high as possible are needed and are used in many countries, e.g., flexibility in recruiting (times available for the interview, second call, if a no show, *etc.*) and awareness of the study by letters, postings, interviews or news at the sampling points. We approached not only these actions to favor participation, but also the opportunity to have an individual feedback by a report at the end of the study which includes diet assessment, physical activity level, anthropometry, and general advice on how to get an adequate EB and modulate its main determinants. In any case, it is deemed important also to collect information on non-respondents in order to eventually correct for differences in response, as the ANIBES Study already has done.

### 4.1. Dietary Assessment Methodologies

Several dietary assessment tools at the individual level are available [3,16,17,23,33,56,66,67,68,69]. In general, these methods can be divided into two basic categories: those that record data at the time of eating (prospective methods, *i.e.*, so-called weighed and estimated records methods including those using new technologies) and those that collect data about the diet eaten in the recent past or over a longer period of time (retrospective methods, interview methods). Interview methods may refer to current diet (24-h food recalls) or habitual diet (dietary history and food frequency method) [70].

### 4.2. 24-h Dietary Recall

During the interview an individual recalls actual food intake for the immediate past 24 or 48 h or for the preceding days. The 24-h dietary recall is the most common recall method used [3,16,66,71,72]. The role of interviewers is crucial in administering 24-h recall interviews because this information is obtained by asking probing questions. Therefore, the previous training is critical for better results and a two full day course was completed by ANIBES interviewers before pilot studies and main fieldwork. Standard procedures advise that no prior notification should be given to the subjects about whether or when they will be interviewed about their food intake. For ANIBES, 24-h dietary recall was carried out during the first visit (face-to-face) by the interviewer without previous knowledge. The repetition, per each subject, of a 24-h recall on non-consecutive days provides consumption data on independent days and, consequently, the estimation of the within-person variability of intake. However, the latter may be skipped if dietary record using new technologies is considered the key tool to collect diet information, but in combination with one 24-h dietary recall, as it was decided in the present study protocol [28,73].

### 4.3. Dietary Record

The dietary record seems to provide a higher level of detail, but this is not always given by the participants unless new easier and more feasible technologies are included to collect the required information. Sometimes, the “field worker” can complete missing details during completeness check [55]. The description of the consumed foods and beverages can be highly detailed and preferably accompanied by photographs of all the items eaten and drunk in and outside home, arriving at the level of the brand name or further, if required. Specific information on food packaging materials can also be obtained, especially if respondents are asked to keep the packaging materials or take photographs [74,75,76]. In contrast, for the recall method the level of details of food description can be limited by the memory of the respondents. Concerning the response burden, it is considered high for the dietary record, as recording must be done at time of consumption. This requirement may lead to non-response bias as a result of overrepresentation of more highly educated individuals who are interested in diet and health. A low response rate might be a particular problem in some population subgroups. In the present study no significant differences were observed when education and economic level factors were included for response *vs.* non-response analysis.

With regard to the applicability of the methods for the different population groups, the tablets devices were easily accepted by the young age groups (>9 years old) and some concerns were expressed by parents for free internet access. However, tablet use was only strictly permitted for ANIBES software with no free access. Some difficulties may exist concerning the reliability of records on out-of-home consumption. In our study, tablet devices were referred by a minor number of participants as too heavy to take it out of home in every eating occasion, and some of the participants also reported self-confidence and lack of privacy issues. With respect to elderly people, the dietary record method is suitable but needs adaptations similar to those needed for children, whereas the recall is considered inappropriate because of the cognitive decline often experienced by a high percentage of this age group [68]. In addition, some help may be needed from family and/or caregivers. As explained in the protocol, we attempted people from all age groups (9–75 years) but only living in households. People living in institutions were excluded. This means that elderly people included in the ANIBES study were able to complete the dietary record either with the tablet (few) or by telephone interview.

It is important to remark that previously this kind of methodology has not been used to enable highly standard dietary records across EU countries, as we have attempted at national level throughout ANIBES. However, software has been available to enable highly standardized 24-h recalls (e.g., the dietary software developed by the European Prospective Investigation into Cancer and Nutrition, EPIC-SOFT) [71].

As for the validity of the methods, the usual eating pattern may be influenced or changed by the recording process and participants might forget to photograph or record some foods and drinks. In order to allow the calculation of the intra-individual variability, it is usually considered that subjects must record consumption for at least three days, such as has been done in the ANIBES Study. The validity of the 24-h recall method mainly depends on the respondents short-term memory. When the recall is announced, subjects can reduce their consumption and produce a bias. The method is also quite highly vulnerable due to variability between interviewers. The accuracy in the quantification of the consumed portion sizes is higher for the dietary record over the 24-h recall but only if these are measured or weighed or by taking photographs. Moderate underreporting may occur for both methods, particularly in some subgroups (e.g., obese persons) [77]. Fortunately, we were able to check the validity of both methods in the present study, for the different age groups (9–12; 13–17; 18–64; 65–75 years), sex, geographical area, habitat size, BMI and other variables. Results may be considered highly satisfactory when both methods are combined.

From a statistical point of view it is more efficient to extend the number of participants rather than the number of days [16,73]. On the other hand, it can be more efficient to include more recording or recall days per person in order to estimate habitual exposure to compounds present in less frequently consumed foods where this was not the main goal of ANIBES Study. Moreover, when using the dietary records technique, accuracy of records may decrease as the number of days increase [55]. In practice, no more than three or four consecutive days should be included because of respondent fatigue [78]. From our study, we may also conclude that fatigue may be a variable to be considered although dietary records with a higher number of days (4 or 5) than initially scheduled (3) have been also reported and specifically treated during the three data cleaning stages, as explained. The missing of potential food/drinks records within a day was more common as a general conclusion from the ANIBES Study.

### 4.4. Interview Options

The main advantages of the face-to-face interview are that it allows for better rapport; the more personal relationship may increase response rate due to personal contact with the interviewer and there is a potential for more detailed probing of participant responses, but it is time-consuming and expensive; the body size of the interviewer and of the subject may be an issue and affect the responses of the participant [79]. In person, the subject may be more vulnerable to exaggerate consumption of foods they perceive to be good and underreport foods perceived to be unhealthy. Therefore, the importance of well-trained interviewers for fieldwork unrelated to the nutrition and dietetics and/or consumer science adds more potential reliability for collection of the data [80]. On the other hand, that strategy must be combined with a second-level careful dietician-nutritionist review and validation of the information recorded as has been done in ANIBES.

The increasing use of the telephone interview as a research method may be a reflection of broader social change and technological advances, with increased use and acceptability of telecommunications [80,81,82,83]. The main advantage of a telephone-administered interview is saving time and therefore saving budget in large surveys. It allows for centralized training and supervision of interviewers and for increased speed of data gathering and processing. Sampling is not geographically restricted so this method is particularly useful where geographical location could be a barrier to face-to-face interviews. At this point it is remarkable that ANIBES was carried out by using 128 local sampling points of the inland of Spain plus Canary and Balearic Islands. In our knowledge, no previous national dietary/physical activity survey was able to attempt the combination of such representative levels, the use of new technologies (tablet devices and related software), and face-to-face visits for anthropometric measurements, physical activity and general questionnaires and 24-h dietary recall. In addition, for those individuals not able to use the tablet device, alternatives were offered: photo camera and paper or telephone interview. In consequence, a broad spectrum of valid instruments and tools were employed in the ANIBES Study. The main drawback of the telephone-administered interview is that the personal touch is lost, but the use of probing techniques by the skilled interviewer can considerably reduce the amount of under-reporting. For best results, a picture booklet may be designed and provided to all participants prior to the telephone call [74,76,83].

New computer-based technologies (*i.e.*, special devices such as tablets, mobile phones or web computer assisted internet) and combinations of qualitative and quantitative methods are now highly recommended for dietary assessments [16,28,74]. Clearly, technology is changing how dietary assessment methods are being delivered. Although web-based assessments are more accessible and enable larger nutritional epidemiological studies to be conducted, validation and calibration methods are needed to fully utilize this new frontier in dietary assessment methodology. Moreover, it is still expected that those new technology-based dietary surveys could be considered as top priority either at country or European level (e.g., EFSA) [16].

The interviews can be administered either by a trained interviewer or by a dietician-nutritionist. However, if trained interviewers are used, then perhaps monitoring and routine checking of their dietary interviews by a registered dietician-nutritionist during the survey period would be important as ANIBES is able to show; on the other hand, computerized dietary assessment is an area of growing interest and several studies [73,84,85,86] have examined the use of computer software to assist in the dietary interview.

One of the main errors that occur while measuring food consumption in dietary epidemiological surveys is the assessment of portion size, both in terms of definition and in accuracy of quantification [87]; hence, measurement tools have to be selected carefully. The methods used to quantify portion size can be divided into two broad categories: those where foods and leftovers are weighed and/or photographed respectively before and immediately after consumption and those where food portions are estimated. Weighing or taking photographs before and after eating is considered to be the most accurate method for measuring food intake. The disadvantages of this method, called “the weighed method”, are that it is time consuming (taking photographs seem to be more feasible), costly and disruptive [88] and there are many circumstances in which scales may not be available. Weighing each food item can also introduce changes in eating habits and similar concern may occur when tablet and/or mobile phone are used for dietary record; in addition, there are circumstances where weighing is not suitable, for example in large epidemiologic studies [32] and new technology as used in our study seems to be best alternative option; moreover, if the out-of-home record wants to be also precisely recorded. In the ANIBES study, we found a very low response during the first pilot study mainly due to the innovative system for collecting the data, some drawbacks with the tested software or the time-consuming concern. However, the decision to create different working discussion (interviewers; potential participants; parents; researchers) after the first pilot study helped to fix problems reported by the responders and non-responders, but also to improve tablet and software use, and in general all the skills to be employed. Those improvements were tested during the second pilot study and afterwards at the main fieldwork where high response was obtained.

There are a number of measurement aids that can be used while estimating food intake which help to avoid common errors in assessment of portion sizes [89]. Such aids, frequently referred to as portion-size measurement aids (PSMAs) including photographs, food models, household measures, *etc.*, have been used separately or in combination in dietary data collection [66,71,90]. In the ANIBES study we were able to use these aids. However, there is also general agreement that no “gold standard’ as such exists for estimation of portion size [91,92], and all established approaches show advantages and disadvantages that have been summarized by Wrieden [92]. Systematic bias and large random error may occur while quantifying foods; therefore, as it is accepted that there is no perfect way of measuring habitual intake, the method selected for each study will depend on several factors, which include convenience for the subject, degree of accuracy required, expense and targeted population [93]. All these variables were taken into account and carefully revised both at the study design period, applied during the two pilot studies and also at the checkup post data collection, as it is highly recommended that a *post hoc* analysis of the observed food consumption data be performed in order to assess uncertainty related to potential under-reporting.

### 4.5. Physical Activity

Techniques for measuring physical activity level (PAL) usually include Heart Rate Monitoring (HRM), motion sensors (accelerometers), as well as self-reporting instruments, such as activity diaries and questionnaires [94,95,96]. It is well recognized that each of these assessment methods has its own associated advantages and limitations [97]. Questionnaires have been often considered the only feasible method for assessing habitual physical activity in large populations, because they are easy to administer, relatively inexpensive, and non-invasive. The other three methods are much more complicated in terms of logistical and financial resources to be included in a large/representative dietary survey. In the present ANIBES Study a combination of an international validated questionnaire (IPAQ) and the use of accelerometers in a subsample (10%) targeted with equal criteria as for the whole sample was approached. However, it must be considered that accurate and reliable assessment of habitual physical activity is particularly challenging if these are of low intensity, not done routinely, *etc.* [98]. Most of the questionnaires available in the literature focus on recreational rather than total activity, probably because it is easier to recall repeated discrete activities that are undertaken for a limited period of time and for which a conscious choice is made prior to engagement, and few questionnaires have been designed or lack use to assess overall physical activity at work, recreation and domestic life [99]. It is acknowledged that the administration of physical activity questionnaires validated in the specific context and/or population is particularly recommended, due to the high degree of specificity of physical activity. Ideally physical activity questionnaires should be validated by comparison with an objective method, such as an accelerometer [97,99]. In the ANIBES study, we were able to cover that priority goal.

### 4.6. Anthropometry

It is well agreed that at least body weight should be recorded as part of a dietary survey [100]. Two methods are generally used to record body weight and height in the context of a food dietary survey, namely “self-reporting by subjects” or “measured by the interviewer”. Unfortunately, the costs of actual measurements are often high, and often also require special training of interviewers for accurate assessment. We attempted to use validated instruments (stadiometers, scales, and measuring tape) to obtain accurate information on height, weight, waist circumference) and further calculations (e.g., BMI or % total body fat).

## 5. Conclusions

The innovative strengths of the design, protocol and methodology used in the ANIBES Study are:

- The first dietary survey in Spain that looked specifically on the “energy balance” paradigm at the population level.
- Included a representative age sample (9–75 years old) of the Spanish population.
- The first study carried out in the same individuals which allow information collection on diet, physical activity, anthropometry and body composition.
- Employed for the first time in Spain new technology to collect information on intake and physical activity (using tablet devices in nearly real time) to avoid the well known and common problems of under/over reporting.
- The new ANIBES software (food and beverage database) allowed the most detailed information (not only by the usual food groups included in food composition tables) to be obtained, also for subgroups which are much more accurate and adjusted to the current “real” food market.
- A precise quantification of physical activity was achievable nationwide (combined use of self-validated questionnaires plus objective accelerometers), to avoid the key problem of underreporting and to evaluate factors such as type, duration, amount, and intensity, which are rarely reported in population nutrition surveys.

The main drawbacks were the difficulties for some participants to use the new technologies, or the lack of seasonality for food collection or measuring physical activity level.

In summary, considering the carefully designed protocol based on best evidence available and previous experience, the ANIBES study may contribute to provide useful data to inform food policy planning, food-based dietary guidelines development and other health-oriented actions.
